# High-density lipoprotein cholesterol as a predictor of poor survival in patients with nasopharyngeal carcinoma

**DOI:** 10.18632/oncotarget.7160

**Published:** 2016-02-03

**Authors:** Yan-Yan Liu, Shao-Jun Lin, Yuan-Yuan Chen, Li-Na Liu, Liu-Bin Bao, Lin-Quan Tang, Jing-Song Ou, Zhi-Gang Liu, Xiao-Zhong Chen, Yan Xu, Jun Ma, Anthony T. Chan, Ming Chen, Yun-Fei Xia, Wan-Li Liu, Yi-Xin Zeng, Hai-Qiang Mai, Mu-Sheng Zeng, Jian-Ji Pan, Xing Zhang

**Affiliations:** ^1^ Sun Yat-Sen University Cancer Center, State Key Laboratory of Oncology in South China, Collaborative Innovation Center for Cancer Medicine, Guangzhou, China; ^2^ Division of Nephrology, Department of Internal Medicine, Tongji Hospital, Tongji Medical College, Huazhong University of Science and Technology, Wuhan, China; ^3^ Department of Radiation Oncology, Fujian Provincial Cancer Hospital Affiliated to Fujian Medical University, Fuzhou, China; ^4^ Department of Radiation Oncology, Zhejiang Cancer Hospital/Zhejiang Key Laboratory, Hangzhou, China; ^5^ Department of Oncology, The Second Affiliated Hospital of Guangzhou Medical College, Guangzhou, China; ^6^ Division of Cardiac Surgery, The First Affiliated Hospital, Sun Yat-Sen University, Guangzhou, China; ^7^ Department of Radiation Oncology, Hunan Provincial Tumor Hospital, Tumor Hospital Xiangya School of Medicine of Central South University, Changsha, China; ^8^ Partner State Key Laboratory of Oncology in South China at Sir YK Pao Centre for Cancer, The Chinese University of Hong Kong, Shatin, Hong Kong

**Keywords:** high-density lipoprotein cholesterol, nasopharyngeal carcinoma, prognosis

## Abstract

**Purpose:**

We aimed to assess the prognostic value of pretreatment high density lipoprotein cholesterol (HDL-C) levels in patients with nasopharyngeal carcinoma (NPC) and investigate the possible biological effects of these lipoproteins on NPC cells *in vitro*.

**Experimental Design:**

We examined the prognostic value of pretreatment HDL-C levels in 2443 patients with non-metastatic NPC from three independent institutions. The Cox proportional hazard model and log-rank test were used to analyze the correlation between HDL-C levels and overall survival (OS). Cell growth, colony formation, and apoptotic assays were used to determine the biological functions of HDL on NPC cells *in vitro*. All of the statistical tests were two-sided.

**Results:**

OS was decreased in patients with high pretreatment HDL-C levels compared with those with low HDL-C levels (*P* < 0.05). Similarly, a decreased OS was noted in advanced stage (stage III-IV), NPC patients with high pretreatment HDL-C levels (*P* < 0.01). Multivariate analyses indicated that HDL-C was an independent prognostic factor associated with shorter OS in training cohorts. These findings were confirmed in both independent validation cohorts (*P* < 0.01). *In vitro* experiments demonstrated that HDL could increase cell proliferation, invasion, and colony formation, which were largely dependent on the expression of its receptor SR-B1. Finally, HDL could enhance chemoresistance by protecting cancer cells from apoptosis.

**Conclusions:**

Pretreatment HDL-C is a poor prognostic factor for patients with NPC. This effect may be associated with the ability of HDL to enhance proliferation, colony formation, migration, and chemoresistance in NPC cells.

## INTRODUCTION

Recently, several epidemiological studies have demonstrated that dyslipidemia is correlated with the risk of breast, colon, and prostate cancers and that altered lipid profiles are associated with cancer development and metastasis [1, 2]. In addition, there is an abundance of data suggesting that dysregulated lipid metabolism might serve as a hallmark of cancer because dysregulated metabolism has increasingly been recognized as a common property of malignant cells [3–5].

HDL has been a source of great fascination in the discipline of lipidology. HDL possesses potent biological activities, including anti-oxidative, anti-inflammatory, and anti-infective activities [6–8]. The role of HDL-C in the development and progression of atherosclerosis has been well illustrated [9–11], but its role in cancer has not been thoroughly investigated. Guo demonstrated that serum HDL-C could serve as a potential biomarker for nodal stages in gastric cancer [12], but Tamburrini noted that there was no evidence indicating any association between dietary and serum cholesterol levels and mammographic density in breast cancer [13].

Nasopharyngeal carcinoma (NPC) is a common squamous-cell carcinoma in certain regions of Asia and Africa, with an age-standardized incidence rate of 20–50 per 100000 males in Southern China [14]. Epstein Bar virus (EBV) infection, genetic and environmental factors, lifestyle, and smoking have been closely correlated with NPC [15–17]. The properties of tumor itself, primary tumor volume and location are the key points for recurrent NPC [18]. Most recently, we have observed that Scavenger Receptor B1 (SR-B1), the major HDL membrane receptor, is overexpressed in NPC cell lines and tumors [19]. Other studies have demonstrated that the expression of SR-B1 is increased in prostate and breast tumors [20] but not in the normal surrounding tissues, suggesting that SR-B1 could be used as a novel cancer biomarker. Given that the major function of SR-B1 is to mediate the selective transfer of cholesteryl ester from HDL molecules to cells, we hypothesized that the serum levels of HDL-C may increase the amount of influx transfer of cholesteryl ester to NPC cells expressing SR-B1, which, in turn, exerts biological effects on cancer cells and leads to differences in the clinical outcome of NPC patients. Thus, our objective was to identify the prognostic value of serum HDL-C and to investigate the biological effects of lipoproteins on NPC cells *in vitro*.

## RESULTS

### Pretreatment HDL-C predicted poor prognosis for NPC patients

**Figure 1:**
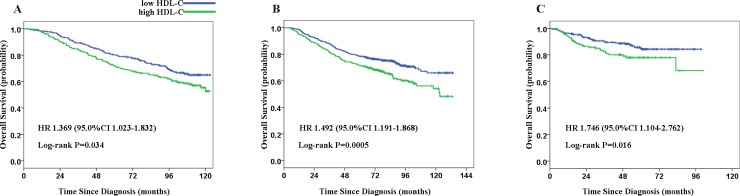
Kaplan-Meier estimate of overall survival (OS) for patients with nasopharyngeal carcinoma (NPC) according to serum HDL-C levels The following analyses are presented: OS (**A**) in 530 patients in the training cohort; OS (**B**) in 982 patients in the validation cohort A; and OS (**C**) in 500 patients in the validation cohort B. *P* values were calculated using the log-rank test.

To investigate the potential effect of serum HDL-C in NPC patients, ROC curve analysis was used to compare the specificity and sensitivity to predict the prognosis of NPC patients. With the cut-off value of 1.295 mmol/L in the training cohort, patients with high pretreatment HDL-C levels exhibited decreased OS (hazard ratio [HR] 1.369 [1.023–1.832]; *P* = 0.034) compared with patients with low HDL-C levels (Figure 1A). In subgroup analysis, HDL-C remained a predictor of NPC prognosis in patients with advanced stage (stage III-IV) NPC. Compared with patients with low HDL-C, patients with high HDL-C levels exhibited a decreased OS (hazard ratio [HR] 1.568 [1.128–2.179]; *P* = 0.007) in the training cohort (Supplementary Figure 2D). However, this association was not observed in patients with early stage (stage I-II) NPC in the training cohort. The cumulative 5-year survival rate was 80.2% in the low HDL-C group, whereas the rate was only 72.0% in the high HDL-C group in the training cohort (Table 1).

**Table 1:** Clinical characteristic of patients according to HDL-C levels in the training and validation cohorts

Characteristic	Training cohort (*n* = 530)		Validation cohort (*n* = 982)		Validation cohort (*n* = 500)	
	low HDL-C group	high HDL-C group	low HDL-C group	high HDL-C group	low HDL-C group	high HDL-C group
Total no· of patients	256 (48.3%)	274 (51.7%)	563 (57.3%)	419 (42.7%)	338 (67.6%)	162 (32.4%)
Age, years						
≤ 45	132 (51.6%)	131 (47.8%)	279 (49.6%)	178 (42.5%)	130 (38.5%)	49 (30.2%)
> 45	124 (48.4%)	143 (52.2%)	284 (50.4%)	241 (57.5%)	208 (61.5%)	113 (69.8%)
Sex						
Male	196 (76.6%)	197 (71.9%)	443 (78.7%)	281 (67.1%)	259 (76.6%)	103 (63.6%)
Female	60 (23.4%)	77 (28.1%)	120 (21.3%)	138 (32.9%)	79 (23.4%)	59 (36.4%)
Pathology						
WHO type II	11 (4.2%)	13 (4.7%)	18 (3.2%)	10 (2.4%)	NA	NA
WHO type III	245 (95.8%)	261 (95.3%)	545 (96.8%)	409 (97.6%)	NA	NA
Tumor stage						
T1	22 (8.6%)	37 (13.5%)	17 (3.0%)	14 (3.3%)	64 (18.9%)	32 (19.8%)
T2	100 (39.1%)	100 (36.5%)	173 (30.7%)	130 (31.0%)	143 (42.3%)	76 (46.9%)
T3	47 (18.4%)	46 (16.8%)	264 (46.9%)	189 (45.1%)	86 (25.4%)	31 (19.1%)
T4	87 (34.0%)	91 (33.2%)	109 (19.4%)	86 (20.5%)	45 (13.3%)	23 (14.2%)
Node stage						
N0	56 (21.9%)	74 (27.0%)	97 (17.2%)	70 (16.7%)	68 (20.1%)	33 (20.4%)
N1	85 (33.2%)	99 (36.1%)	205 (36.4%)	143 (34.1%)	114 (33.7%)	65 (40.1%)
N2	107 (41.8%)	92 (33.6%)	209 (37.1%)	173 (41.3%)	116 (34.3%)	51 (31.5%)
N3	8 (3.1%)	9 (3.3%)	52 (9.2%)	33 (7.9%)	40 (11.8%)	13 (8.0%)
TNM stage						
I	8 (3.1%)	10 (3.6%)	4 (0.7%)	2 (0.5%)	18 (5.3%)	8 (4.9%)
II a–b	58 (22.7%)	87 (31.8%)	85 (15.1%)	56 (13.4%)	83 (24.6%)	50 (30.9%)
III	94 (36.7%)	78 (28.5%)	319 (56.7%)	252 (60.1%)	159 (47.0%)	68 (42.0%)
IV a–b	96 (37.5%)	99 (36.1%)	155 (27.5%)	109 (26.0%)	78 (23.1%)	36 (22.2%)
Overall survival						
Deaths	78 (30.5%)	108 (39.4%)	153 (27.2%)	152 (36.3%)	43 (12.7%)	32 (19.8%)
5-year	80.2%	72.0%	78.5%	71.4%	83.60%	76.20%

Data are *n* (%) or median (IQR). NA = not information available. * χ^2^ test.

In the independent validation cohort A, similar results were achieved when the same cut-off value was applied (Figure 1B). We observed that patients with low HDL-C levels exhibited favorable survival compared with patients with high serum levels of HDL-C (hazard ratio [HR] 1.492 [1.191–1.868]; *P* = 0.0005). The cumulative 5-year survival rate was 78.5% in the low HDL-C group but 71.4% in the high HDL-C group in the validation cohort (Table 1). When we analyzed subgroups (stage I-II and stage III-IV), serum HDL-C was identified as a prognostic marker in both groups (stage I-II, *P* = 0.020; stage III-IV, *P* = 0.005, Supplementary Figure 2B and 2E). To confirm the results, we also assessed the survival statuses with another independent validation cohort (cohort B) of NPC patients from a non-endemic geographical region. Similarly, patients with high HDL-C levels exhibited lower OS rates compared with those with lower HDL-C levels (hazard ratio [HR] 1.746 [1.104–2.762]; *P* = 0.016, Figure 1C). Consistent with the observation in the training cohort, the prognostic value of HDL-C was only observed in patients with advanced stage (stage III-IV) NPC (hazard ratio [HR] 1.892 [1.154–3.102]; *P* = 0.01, Supplementary Figure 2F) in the subgroup analysis.

### Pretreatment HDL-C is an independent prognostic marker for NPC patients

To determine whether serum HDL-C is an independent prognostic factor, we applied multivariable analysis. We measured the age, T classification, N classification, and HDL-C levels because they were all significantly correlated with survival in the Kaplan-Meier analysis and log-rank test in the training cohort (for HDL-C, *P* = 0.035; for age, T classification, and N classification, *P* < 0.01; Supplementary Table). Cox models were developed with survival measured since NPC diagnosis as the endpoint in the training and validation cohorts (Table 2). Consistent with the univariate analysis results, the pretreatment serum HDL-C level was identified as an independent prognostic factor for overall survival in all cohorts (*P* = 0.015, 0.001, and 0.006). In addition, age, T stage, and N stage were also identified as independent factors for the prognosis of NPC patients in multivariable analysis.

**Table 2:** Summary of multivariate analyses of prognostic factors in the training and validation cohort

Endpoint	Training Cohort		Validation Cohort		Validation Cohort	
	HR (95% CI)	*P* value	HR (95% CI)	*P* value	HR (95% CI)	*P* value
Death						
Sex, male vs female	0.915 (0.652–1.285)	0.609	0.784 (0.596–1.031	0.081	1.012 (0.597–1.714)	0.965
Age, ≥ 45 years vs < 45years	2.069 (1.527–2.802)	< 0.001	1.577 (1.248–1.991)	< 0.001	3.090 (1.692–5.644)	< 0.001
WHO pathological type, type III vs type II	1.436 (0.701–2.942)	0.323	0.791 (0.555–1.129)	0.197	NA	NA
T classification, T3–4 vs T1–2	1.638 (1.217–2.206)	0.001	1.591 (1.235–2.051)	< 0.001	3.103 (1.933–4.980)	< 0.001
N classification, N2–3 vs N0–1	1.732 (1.282–2.339)	< 0.001	2.060 (1.634–2.596)	< 0.001	1.963 (1.227–3.139)	0.005
HDL-C, mmol/L (≥ 1.295 vs < 1.295)	1.443 (1.075–1.936)	0.015	1.451 (1.155–1.822)	0.001	1.934 (1.213–3.083)	0.006

### The biological effects of HDL on cancer cells *in vitro*

Given the adverse prognostic value of serum HDL-C levels in NPC patients in this three-cohort independent retrospective study, we proceeded to investigate the biological effects of HDL on cancer cells *in vitro*. To eliminate the potential effects of bovine serum HDL, we used a serum-free culture medium (Keratinocyte-SFM). Keratinocyte-SFM is a complete serum-free medium supplemented with human recombinant Epidermal Growth Factor (rEGF) and Bovine Pituitary Extract (BPE), which has been successfully used to support the growth of primary nasopharyngeal epithelial cells and nasopharyngeal carcinoma cells [21, 22].

**Figure 2:**
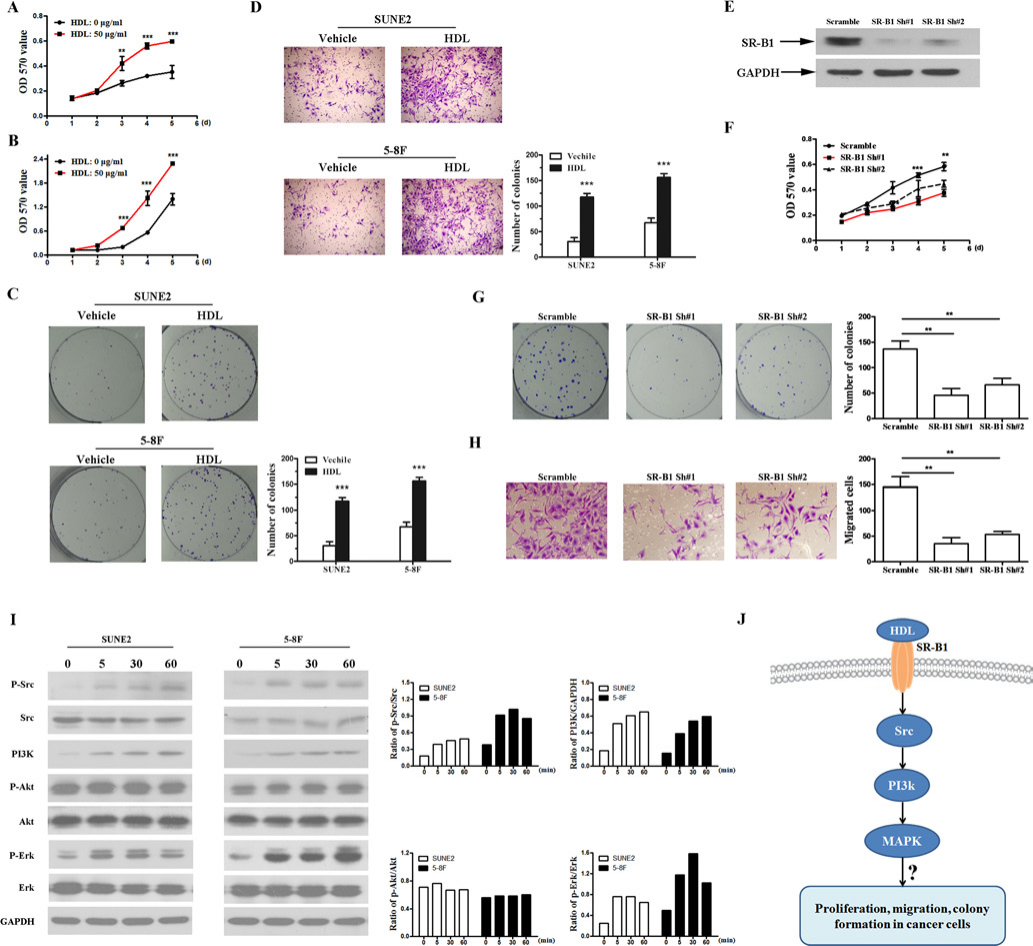
HDL promotes proliferation and enhances the transformation ability of NPC cells The effects of HDL (50 μg/mL) on SUNE2 (**A**) and 5-8F (**B**) cell proliferation were determined by MTT assay. (**C**) Colony formation assay was performed on SUNE2 and 5-8F cell lines cultured with medium alone or HDL at 50 μg/mL for 6 days. (**D**) A Boyden chamber assay was performed. SUNE2 and 5-8F cells were plated on the upper cell culture inserts, and the culture medium was plated in the lower chambers in the presence or absence of HDL at 50 μg/mL for 24 h. Original magnification, ×400. (**E**) The expression of SR-B1 in SR-B1 shRNA (shRNA#1, shRNA#2) or scramble shRNA-expressing SUNE2 cells was analyzed using Western blot. (**F**) The effects of HDL (50 μg/mL) on SR-B1 shRNA or scramble shRNA cell proliferation were determined by MTT assay. (**G**) A colony formation assay of SR-B1 shRNA or scramble shRNA cells cultured with HDL at 50 μg/mL for 6 days was performed. (**H**) A Boyden chamber assay was performed. SR-B1 shRNA or scramble shRNA cells were plated on the upper cell culture inserts, and the culture medium was plated in the lower chambers in the presence of HDL at 50 μg/mL for 24 h. Original magnification, ×400. (**I**) The cells were incubated with HDL (50 μg/mL) for the indicated time periods, and extracts were analyzed by Western blot. The ratios of phospho-Src to Src (p-Src/Src), PI3K to GAPDH (PI3K/GAPDH), phospho-AKT to AKT (p-AKT/AKT) and phospho-ERK to ERK (p-ERK/ERK) were calculated after densitometric analysis was performed on the Western blots. (**J**) A schematic representation of the major molecular mechanism of the biological effects of HDL on NPC cells. Error bars represent SEM. *P < 0.05, **P < 0.01 and ***P < 0.001 compared with the cells treated with medium alone.

The optimal HDL concentration (50 μg/mL) was determined by MTT assay [7, 23] (Supplementary Figure 1). As shown in Figure 4, increased cell growth was observed in both cell lines after 3 days of culture in HDL-containing media. The population-doubling time of each cell line with HDL was significantly shorter compared with the control cell lines (Figure 2A–2B, *P* < 0.05). Meanwhile, we performed a colony formation assay to investigate whether HDL can enhance the transforming ability of NPC cells. The results demonstrated that there were more and larger colonies of NPC cells treated with HDL than those formed by the control cells without HDL (Figure 2C, *P* < 0.001). Subsequently, Boyden chambers coated with Matrigel were used to examine the invasive ability of NPC cells. Compared with NPC cells in Keratinocyte-SFM alone, the number of migrating cancer cells upon culturing for 24 h with the addition of HDL significantly increased 2- to 3-fold (Figure 2D, *P* < 0.001).

Previously, we reported that SR-B1, the HDL receptor, was overexpressed in NPC cell lines and tumors. To examine whether the observed functions of HDL is dependent on the expression of its receptor (SR-B1) in cancer cells, we silenced SR-B1 in SUNE2 cells using specific shRNAs. Both shRNA #1 and shRNA #2 efficiently knocked-down endogenous SR-B1 expression (Figure 2E). As shown in Figure 2F–2H, silencing endogenous SR-B1 in SUNE2 cells markedly repressed cell proliferation, colony formation, and the invasion of NPC cells under stimulation with HDL. In cells cultured without HDL or with 10% FBS, we did not observed any obvious repression of cell growth in SUNE2 cells following the inhibition of endogenous SR-B1 (data not shown). Therefore, these data indicate that the biological effects of HDL on NPC cells are largely dependent on the expression of its receptor, SR-B1.

HDL functions as a signaling molecule by initiating AKT and MAPK/ERK signaling pathways to protect vascular endothelial cells [24–26]. To better understand the mechanisms underlying HDL-related effects on NPC cells, we determined whether the AKT and MAPK signaling pathways were activated. Src kinase, PI3K, AKT, and ERK activity was measured by Western blot in NPC cells incubated from 5 to 60 min with HDL. As shown in Figure 2I, HDL activated PI3K and stimulated the phosphorylation of Src and ERK but not AKT. Enhanced phosphorylation was detected as early as 5 min and reached maximal phosphorylation at 30 min. These data suggest that HDL could stimulate Src kinase, which in turn activates the PI3K and MAPK/ERK pathways (Figure 2J).

### HDL increases the chemoresistance of NPC cells

**Figure 3:**
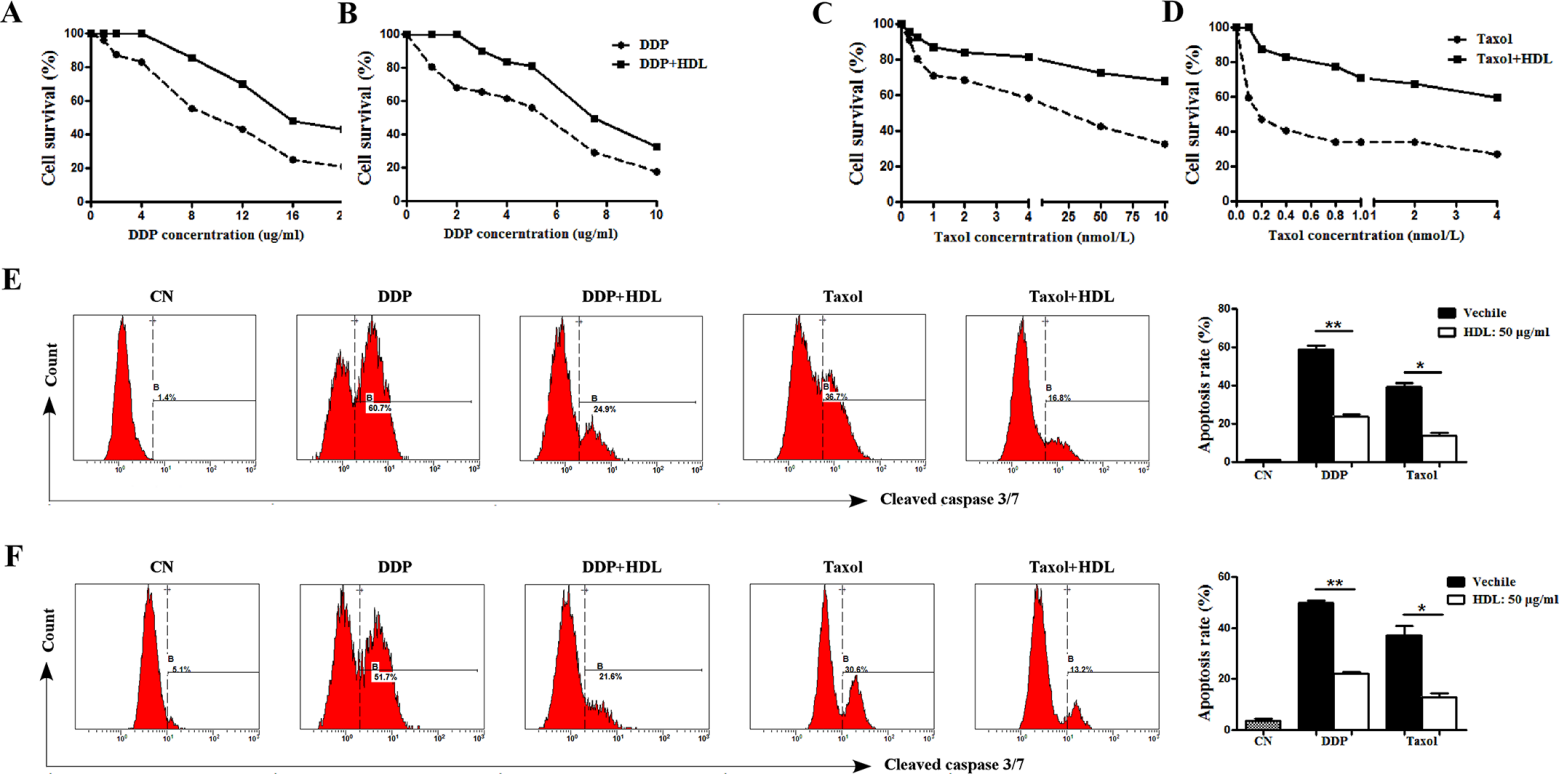
HDL increases the chemoresistance of NPC cells The growth curves of SUNE2 (**A**, **C**) and 5–8F (**B**, **D**) cells 48 h post-treatment with the indicated doses of DDP (the right panel) and Taxol (the left panel). DDP (2 μg/mL) and Taxol (1 μg/mL)-induced apoptosis was assessed in SUNE2 (**E**) and 5–8F (**F**) cells in the presence or absence of HDL at 50 μg/mL. Error bars represent SEM. *P < 0.05, **P < 0.01 and ***P < 0.001.

Given that the most significant prognostic effect of HDL-C was observed in the subgroup containing advanced-stage patients and that most of those patients were receiving concurrent chemo-radiation therapy, we then tested whether HDL reduced the chemosensitivity of NPC cells. For this purpose, NPC cells were treated with two different first-line drugs used for NPC patients in combination with or without HDL. Cell viability was measured 48 h after treatment with increasing concentrations of DDP and Taxol, which revealed that HDL significantly reduced the chemosensitivity of cancer cells exposed to drugs compared with control cells (Figure 3A–3D, *P* < 0.001). These data indicate that elevated HDL levels in the environment of cancer cells decrease their cytotoxic responses to chemotherapeutic agents.

Previous reports demonstrated that serum HDL can protect endothelial cells from apoptosis in the setting of atherosclerosis, and defects in the regulation of apoptosis contribute significantly to chemoresistance. To dissect the mechanism involved, we determined whether chemoresistance induced by elevated HDL levels might be the consequence of a reduction in apoptosis. A caspase-3 and −7 apoptosis assay demonstrated that DDP (2 μg/mL) and Taxol (1 μg/mL)-induced SUNE2 and 5–8F cell apoptosis was attenuated in cells exposed to HDL, revealing the protective role of HDL against chemotherapy in NPC cells (Figure 3E–3F, *P* < 0.001). Collectively, the results indicated that HDL mediates chemoresistance by protecting cancer cells against drug-induced apoptosis.

## DISCUSSION

Traditional opinion holds that HDL-C is the ‘good cholesterol’ [27]. Intriguingly, our study demonstrates that the NPC patients with high HDL-C levels exhibit poor overall survival compared to patients with low HDL-C levels. Consistently, we demonstrated that isolated human HDL could increase proliferation, colony formation, and invasion as well as chemoresistance in NPC cells *in vitro*.

Here, our study suggests that serum HDL-C levels could serve as a novel prognostic marker in NPC patients. EBV-DNA, microRNA signatures, Bmi-1, and the NPC-SVM classifier [28–30] have been reported as valuable predictors in NPC patients [31–33], but it remains difficult to identify uniform criteria among different institutions, and it is also difficult to use as a routine test in clinical practice due to either relatively higher costs or complicated test procedures. Unlike these biomarkers, the assessment of serum HDL-C is cost-effective, reproducible, and routinely performed in clinical practice globally and exhibits a low dependence on operator expertise. Thus, pretreatment HDL-C levels may represent an attractive potential biomarker for the prognosis of patients with NPC both in high-risk groups and non-endemic regions.

To our knowledge, this represents the first study in the field of HDL and cancer biology. HDL-C level were relatively low in NPC patients compared with health controls (data not shown), which was consistent with previous reports that serum HDL-C levels in cancer patients were decreased with respect to healthy volunteers [34–36]. However, in NPC patients, higher serum HDL-C levels predicted poor patient prognosis, which is contradictory to a previous report in gastric cancer. This report demonstrated that HDL-C was a favorable prognostic marker in gastric cancer [12]. The explanation for this discrepancy might be that expression levels of SR-B1 vary or there are different pathways activated by HDL-C in different tissues. We demonstrated here that the *in vitro* effects of HDL were largely dependent on the expression of SR-B1 in NPC cells. This explanation was also supported by our recent finding that SR-B1 was overexpressed in NPC cell lines and NPC tissues. Although, the expression of SR-B1 in gastric cancer requires further study. Moreover, our results also suggested that the binding of HDL to SR-B1 on cancer cells resulted in the activation of Src, and the subsequent activation of the PI3K and MAPK/ERK pathways, indicating that these are the signals involved in the biological function of HDL in cancer cells. However, the crucial factor in HDL signaling, which is responsible for activating the HDL signaling pathway and promoting NPC progression, remains to be determined.

As first-line treatment drugs, DDP and Taxol have been associated with drug resistance and cross-resistance with other chemotherapy drugs. Recent studies revealed that sphingosine 1-phosphate (S1P) plays an important role in chemoresistance [37–39]. The major carrier of S1P in plasma is HDL, and plasma S1P levels positively correlate with HDL levels [40]. In our present study, we observed that HDL mediated chemoresistance by protecting NPC cells from DDP- and Taxol-induced apoptosis. In addition, we have demonstrated that NPC patients with high HDL-C levels exhibit poor overall survival compared to the population with low HDL-C levels, suggesting that the possible mechanism for NPC progression is plasma HDL carrying S1P to alter tumor cell survival under apoptotic stress by chemotherapeutic agents. In future studies, we will identify the protective anti-apoptotic effect of S1P and explore the mechanisms in NPC.

In conclusion, our results suggest that pretreatment HDL-C levels might be a potential biomarker for NPC prognosis, and HDL-C levels could be a useful marker for personalized therapy. To date, oncologists have been largely unaware of the value of pretreatment HDL-C levels in cancers. This is the first study to analyze the potential prognostic value of pretreatment HDL-C levels in NPC, and its findings contradict the current belief that high serum HDL-C levels are a protective factor. A solid understanding of HDL function in cancers may translate directly to patient benefit through lifestyle modification and the implementation of therapeutic strategies to reduce cancer risk and progression.

## METHODS

### Patients

**Figure 4:**
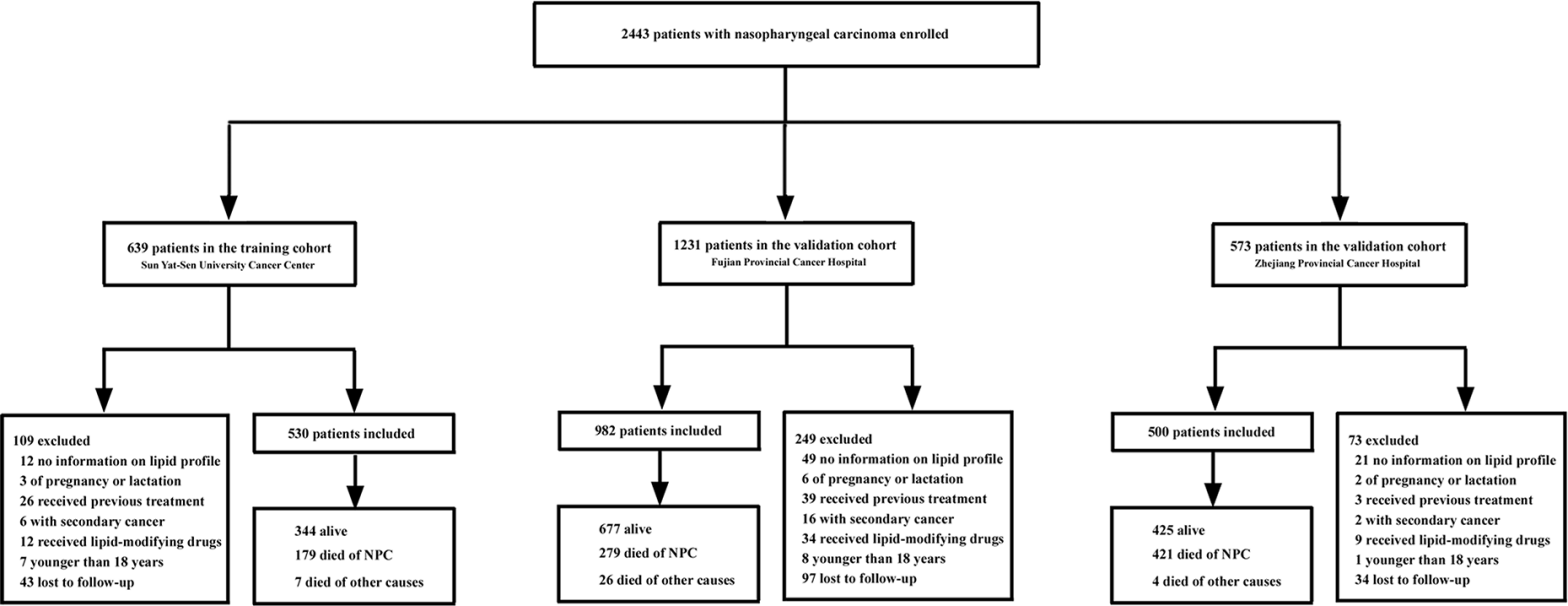
Study profile

A total of 2443 patients with non-metastatic primary NPC were recruited from three independent institutions. From January 2002 to April 2003, 639 patients were enrolled at the Sun Yat-Sen University Cancer Center as the training cohort; 1231 patients were enrolled at Fujian Provincial Cancer Hospital from January 2002 to April 2007 as the validation cohort A; and 573 patients were enrolled at the Zhejiang Cancer Hospital from January 2004 to December 2005 as the validation cohort B. The screening process and detailed information is presented in Figure 4. The final analysis included 2012 primary NPC patients in the training and validation cohorts. The characteristics of the patients in the training and validation cohorts are presented in Table 1. The median follow-up was 95.2 months (IQR 51.6–110.6) in the training cohort and 76.6 months (IQR 61.3–91.8) and 51.8 months (IQR 29.2–63.5) in the validation cohorts A and B, respectively. Our protocol was approved by the institutional review board or ethics committee at each center. Written informed consent was obtained from all patients.

### Assessment of clinical outcomes and patient follow-up

Our primary endpoint was overall survival (OS), and we calculated OS from the date of the first diagnosis of NPC to the date of death from any cause or patient censoring at the date of the last follow-up. After treatment was completed, the patients were evaluated at 3-month intervals for the first 3 years and every 6 months thereafter.

### Selection of the cut-off value in the training cohort

The receiver operating characteristic (ROC) curve analysis was used to select the HDL-C cut-off points for OS in the training cohort. Briefly, the sensitivity and specificity for the outcome observed at each test variable point were plotted to generate an ROC curve. The optimum value located closest to the point (0.0, 1.0) on the curve at maximum sensitivity and specificity was selected as the optimum cut-off value with the greatest discriminatory power for prognostic factors. The optimum score was continuously confirmed in the validation cohort. To use the ROC curve analysis, the patient outcome features were differentiated with respect to the probability of survival (death versus other outcome [censored, alive]).

### Eligibility criteria, staging and treatment plan, and lipoprotein measurements are included in the supplementary

#### *In vitro* study

The *in vitro* details of cell culture, Western blotting, high-density lipoprotein derivation, colony formation assays, invasion assays, and cell proliferation and apoptosis assays are all presented in the Supplementary.

### Statistical analysis

All of the results were expressed as means ± SD, median and IQR, or as n (%) where appropriate. The patients were stratified into two groups according to the targeted lipid and lipoprotein associated with the prognosis. Kaplan-Meier survival estimates were plotted over the time-to-event data, and the log-rank tests for trends were used to assess the association between risk categories and the primary endpoint. Multivariable analyses were performed using the adjusted Cox proportional hazards model to calculate the hazard ratios (HRs) with 95% confidence intervals (CI) and to test the independent statistical significance of the variables. Covariates included age, sex, WHO pathological type, tumor factors, lipid profiles, and smoking status. All statistical tests were two-sided, and *P* values of less than 0.05 were considered statistically significant. The analyses were performed using the statistical test software package SPSS 17.0 and Stata (Version 11).

## SUPPLEMENTARY MATERIALS FIGURES AND TABLES


