# Supplementary material for: High-density lipoprotein cholesterol as a predictor of poor survival in patients with nasopharyngeal carcinoma
Source: Oncotarget. 2016 Feb 3;7(28):42978–87. doi: 10.18632/oncotarget.7160 (PMC5190001; doi:10.18632/oncotarget.7160)
Supplement: Supplementary file 1 [file oncotarget-07-42978-s001.pdf]

High-density lipoprotein cholesterol as a predictor of poor survival in patients with nasopharyngeal carcinoma

Supplementary Materials

Eligibility criteria

The therapeutic eligibility criteria used in this study were as follows: (1) biopsy-proven primary NPC with no radiotherapy, chemotherapy or oncologic surgery history and an ECOG of 0 to 2; (2) at least 18 years of age; (3) adequate haematological, renal, and hepatic function (white blood cell count ≥ 4000/μL, platelet count ≥ 100000/μL, serum creatinine clearance ≥ 50 mL/min, total serum bilirubin concentration < 1.5 mg/dL); (4) complete lipid profile examinations; and (5) available follow-up data. Patients were excluded from this study if they met the following conditions: (1) uncontrolled infection or any previous anticancer therapy or lipid-modifying drugs; (2) pregnancy and lactation or prior malignancy; (3) unsuitable for chemotherapy as a result of a liver, kidney, lung, or heart deficiency; and (4) a history of previous or synchronous malignant tumours.

Staging and treatment plan

According the practice guideline at most Chinese cancer hospitals, all patients with malignancy were submitted to routine blood biochemical examinations, which included lipid measurements. Only 12 (1.89%) patients treated at the Sun Yat-sen University Cancer Center lacked sufficient information from the lipid profile. The oncologists also evaluate the patients' whole-body status using the information from pretreatment routine tests before designing a treatment plan for every patient. In this study, we merely focus on the pretreatment lipid profiles, which were generally analysed 10 to 14 days prior to treatment.

The routine staging work-up included fiberoptic nasopharyngoscopy; computed tomography (CT) or magnetic resonance imaging scans from the suprasellar cistern to the collarbone; clinical examinations of the head and neck region; and a conventional work-up including chest radiography, whole-body bone scan, abdominal sonography or whole body PET/CT. The cancer stage was defined according to the sixth American Joint Committee on Cancer (AJCC) TNM staging manual. All of the patients were treated with conventional, three-dimensional conformal (3DCRT) or IMRT radiotherapy, in accordance with the treatment policy adopted by each institution. The cumulative radiation doses were 66 Gy or greater to the primary tumour and 60–70 Gy to the involved neck area. Bilateral cervical lymphatics and all potential sites of local infiltration were irradiated at 50 Gy or greater. Approximately 436 (82.3%) of the 530 patients in the training cohort and 787 (80.1%) of the 982 patients in the validation cohort received radiotherapy with platinum-based chemotherapy.

Lipoprotein measurements

The lipid and lipoprotein characteristics of NPC patients were determined in the fasting state. All blood samples from NPC patients were obtained before any clinical treatment. Serum TC and TG levels were quantitatively detected using a colorimetric method, HDL-C was measured using the antibody block method, and LDL-C was measured using an enzymatic selective protection method. ApoAI and apoB were determined with turbidimetric methods. All measurements were performed on an automatic clinical chemistry analyser using dedicated kits (Hitachi, 7600–020, Tokyo, Japan).

Cell culture

The poorly differentiated NPC cell lines SUNE2 [22] and 5–8F were maintained in keratinocyte/serum-free medium (Invitrogen) supplemented with recombinant human epidermal growth factor (5 ng/mL), bovine pituitary extract (50 μg/mL), 100 μg/mL streptomycin, and 100 U/mL penicillin G. All incubations were performed at 37°C in a humidified 5% CO_2 atmosphere.

Establishment of SR-B1 stable knockdown cell lines

The poorly differentiated nasopharyngeal carcinoma cell line (SUNE2) was chosen for stable knockdown of SR-B1 expression. Retroviruses were produced performed according to the instructions; SUNE2 cells were infected with the retroviral vector (pSUPER.retro.puro) (Oligoengine) containing SR-B1 short-hairpin RNAs (shRNAs) or empty vector. SUNE2 cells expressing SR-B1 shRNA or empty vector were selected with 0.3 μg/mL puromycin (Sigma) after infection until all of the cells in the non-transfected control culture were killed. At this point, the successful knockdown of the SR-B1 stable cell lines and empty vector stable cell line were verified by western blot and then cultured in

fresh medium. The sequences for the SR-B1 shRNAs were as follows: CCATGACCCTGAAGCTCAT and GCTGAGCCTCTACATGAAA.

Isolation of high-density lipoprotein

HDL was isolated from pooled samples of human plasma using sequential density ultracentrifugation (density = 1.063–1.21 g/mL), as previously reported [23]. The HDL fraction was dialysed against PBS for 72 h with 3 changes per day at 4°C. The protein concentration was measured in triplicate using the BCA Protein Assay Kit. (Thermo, Rockford, USA)

Western blotting

The protein concentrations were quantified by the BCA protein assay kit (PIERCE, Rockford, IL), and 20 µg of protein was used for gel loading. GAPDH primary antibody (Mouse, Santa Cruz, USA) was used at a dilution of 1:3000, and SR-B1 primary antibody (Rabbit, Novus Biologicals) was used at a dilution of 1:2000. The secondary antibody was used at a dilution of 1:3000. Western blotting analysis was performed as previously described [19]. The signals were detected using enhanced chemiluminescence (ECL) (Amersham Pharmacia Biotech, Piscataway, NJ).

Colony formation assay

Cells were collected, counted, and plated in triplicate at 200 cells per well in 6-well plates. The cells were cultured in KSF medium with or without HDL (50 µg/mL), as previously reported [22]. After 7 days, the colonies were visualised and counted after staining with 0.005% crystal violet. The colony formation rate was calculated as the colony number/ number of plated cells. All experiments were performed in triplicate.

Invasion assay

Approximately 2×10^5 cells were plated into the upper chamber of 8-µM pore transwells overlaid with 50 µL of diluted matrigel (BD) solution for invasion. Media containing HDL (50 µg/mL) or vehicle were used as a chemo-attractant in the lower chamber. Cells were allowed to invade the matrigel for 24 h, and the invaded cells were fixed in 1% paraformaldehyde, stained with crystal violet (0.005%), counted in 5 random fields per membrane under phase-contrast microscopy, and averaged.

Cell proliferation

Cells were seeded into 96-well plates (2×10^3 cells per well) and allowed to attach for 6 h. HDL (50 µg/mL) or drugs were subsequently added for another 24, 48, 72, 96, or 108 h. To quantify the amount of metabolically active cells in various groups, 10 µL MTT [3-(4, 5-dimethylthiazol-2-yl)-2, 5-diphenyl tetrazolium-bromide] solution (5 mg/mL in PBS) was added after incubation at 37°C for 4 h. Subsequently, cells were lysed by replacing the media with 100 µL DMSO and dissolved at 37°C for 30 min. The OD was measured at 570 nM using a Spectra Max M5 microplate reader (Molecular Devices, Sunnyvale, CA). The inhibitory concentration of DDP or Taxol was calculated using a relative survival curve.

Apoptosis assay

Approximately 4×10^5 cells per well were plated into 6-well plates. After 24 h, Taxol (1 µg/mL) and DDP (2 µg/mL) was added with or without HDL (50 µg/mL) for another 24 h. The cells were collected and evaluated by flow cytometry after staining with FLICA reagent according to the manufacturers' protocols. The Vybrant FAM Caspase-3 and -7 Assay Kit was purchased from Molecular Probes (Invitrogen). Data were analysed using FlowJo software.

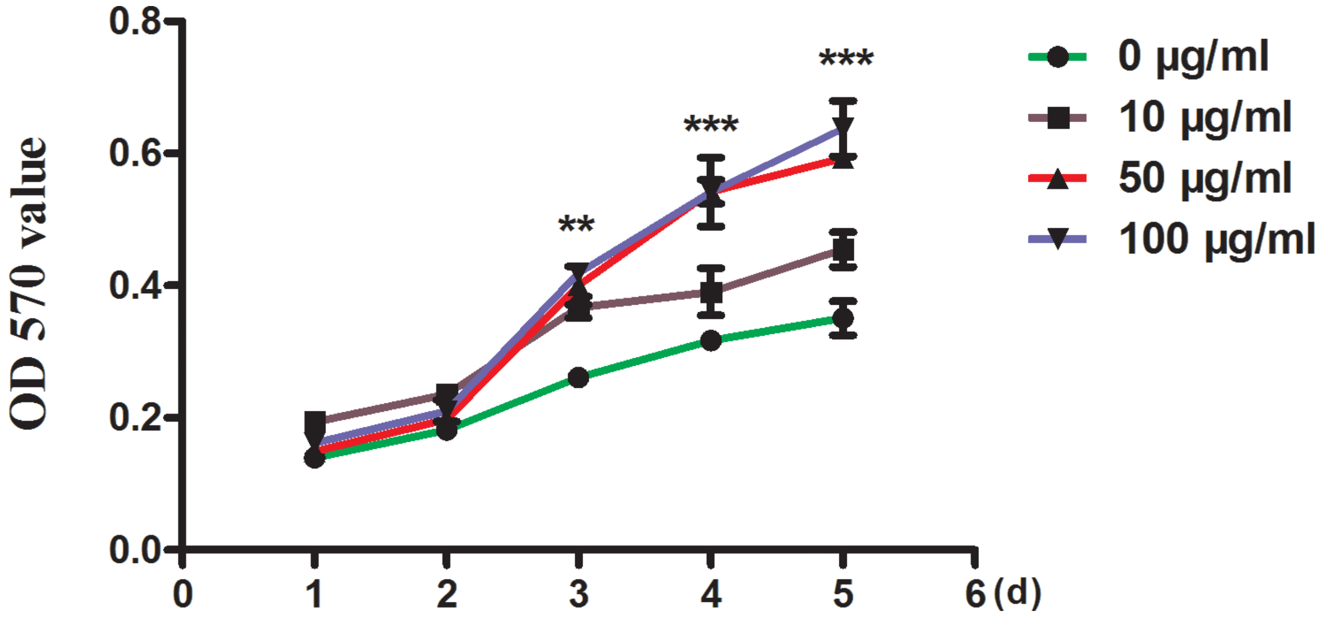

Supplementary Figure S1: HDL promotes NPC cell proliferation. SUNE2 cell proliferation was determined using an MTT assay in the presence or absence of HDL at 10 µg/mL, 50 µg/mL, and 100 µg/mL.

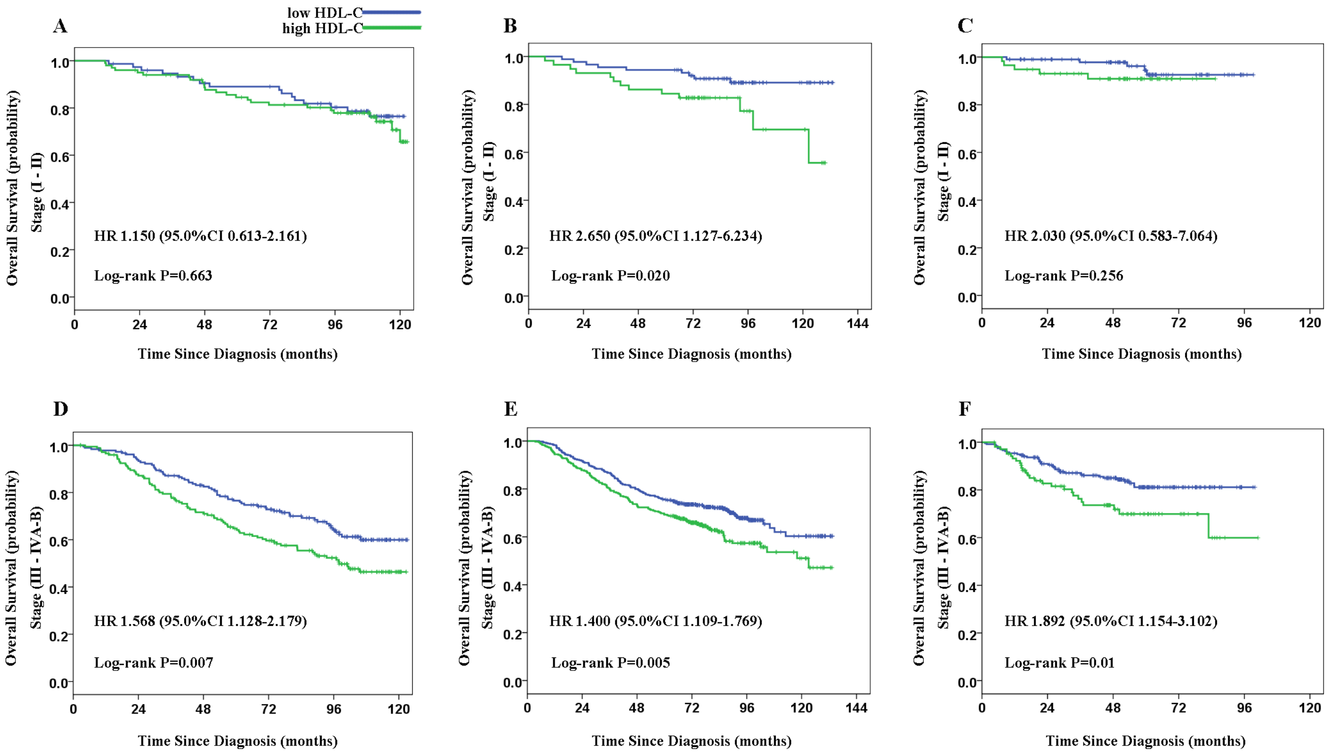

Supplementary Figure S2: Kaplan-meier estimate of OS in NPC patients with early- and advanced-stage disease according to serum HDL-C levels. The following analyses are presented: OS (**A**) in 163 patients with early-stage disease in the training cohort, OS (**B**) in 207 patients with early-stage disease in training cohort A, OS (**C**) in 159 patients with early-stage disease in validation cohort B, OS (**D**) in 367 patients with advanced-stage disease in the training cohort, OS (**E**) in 835 patients with advanced-stage disease in validation cohort A and OS (**F**) in 341 patients with advanced-stage disease in validation cohort B. *P* values for trends were calculated with the log-rank test.

Supplemental Table S1: Univariate analysis of prognostic factors in patients with NPC in the training cohort

Variable	B	HR (95% CI)	*p* value
Sex, male vs female	−0.150	0.861 (0.615–1.204)	0.38
Age, ≥ 45 years vs < 45years	0.683	1.979 (1.468–2.669)	< 0.001
WHO pathological type, type III vs type II	0.179	1.196 (0.589–2.429)	0.62
T classification, T3–4 vs T1–2	0.632	1.882(1.398–2.532)	< 0.001
N classification, N2–3 vs N0–1	0.423	1.527 (1.144–2.039)	0.004
HDL-C, mmol/L (≥1.295 vs < 1.295)	0.314	1.369 (1.023–1.832)	0.035
